# Drinking-Water and Dental Caries

**Published:** 1914-05-02

**Authors:** 


### RESEARCH AND REMEDIES.

### Drinking-Water and Dental Caries.

The attention which dental surgeons and doctors have been devoting in late years to the causes of decayed teeth has resulted in many valuable observations on the best preventive methods. In a recent communication to the *Lancet* Dr. J. B. Cook has investigated a factor which seems to have escaped notice until quite lately—namely, the nature of the drinking-water. As a result of an exhaustive collective inquiry into the dental condition of town children, he finds a distinct correlation between the "hardness" of their drinking-water supply and the prevalence of dental caries: the harder the water, he puts it, the better the teeth. This is not very difficult to understand, since the hardness of drinking-water is mainly due to salts of lime, which are so necessary for the due development of bones and teeth. A further analysis of his statistics revealed another result, which is in accordance with expectations—namely, that the harder the water the lower the infantile mortality, traceable most likely to the superior state of the teeth in those districts where the water is hard. Dr. Cook emphasises the fact that his returns deal solely with town children. He also points out that other factors are not excluded as causes of decay in children's teeth, and that they may have even greater influence.

